# Emotion recognition misclassification patterns in individuals with psychotic spectrum disorders and history of interpersonal aggression

**DOI:** 10.1038/s41537-026-00776-5

**Published:** 2026-06-24

**Authors:** Gabriela Gavalova, Petri Laukka, Lennart Högman, Malin V. Källman, Marianne Kristiansson, Märta Wallinius, Håkan Fischer, Anette G. M. Johansson

**Affiliations:** 1 Department of Clinical Neuroscience, Karolinska Institutet, Stockholm, Sweden; 2 Forensic Psychiatry Unit, Uppsala University Hospital, Uppsala, Sweden; 3 Centre for Psychiatry Research, Karolinska Institutet, Stockholm, Sweden; 4 Department of Psychology, Uppsala University, Uppsala, Sweden; 5 Department of Psychology, Stockholm University, Stockholm, Sweden; 6 Evidence-Based Forensic Psychiatry, Department of Clinical Sciences Lund, Psychiatry, Lund University, Lund, Sweden; 7 Center of Ethics, Law and Mental Health, Institute of Neuroscience and Physiology, The Sahlgrenska Academy, University of Gothenburg, Gothenburg, Sweden; 8 Research Department, Regional Forensic Psychiatric Clinic, Växjö, Sweden; 9 Aging Research Center, Department of Neurobiology, Care Sciences and Society, Karolinska Institutet, Stockholm, Sweden; 10 Department of Behavioural Sciences and Learning, Division of Psychology, Linköping University, Linköping, Sweden; 11 Stockholm University Brain Imaging Centre (SUBIC), Stockholm University, Stockholm, Sweden; 12 Ramsay Clinic Caloundra, Caloundra, QLD Australia

**Keywords:** Schizophrenia, Psychosis

## Abstract

Research suggests a need for more ecologically valid assessments of emotion recognition in aggressive individuals with psychotic spectrum disorder (PSD). We employed a task featuring dynamic, multimodal (visual and auditory) expressions of a broad range of positive and negative emotions. Emotion recognition accuracy and misclassification patterns were compared between individuals with PSD and a history of interpersonal aggression (PSD+AGG; *n* = 79), individuals with PSD without such a history (PSD-AGG; *n* = 72), and healthy controls (HC; *n* = 86). Analyses of variance investigated effects of presentation modality (visual, auditory, multimodal), emotion category (12 emotions), valence (positive, negative), and arousal (high, low). Across analyses, the PSD+AGG group showed significantly lower accuracy than the PSD-AGG group, which in turn showed significantly lower accuracy than the HC group. Misclassification patterns revealed that the PSD+AGG group was more likely to misclassify negative emotions as positive emotions compared to the PSD-AGG group. Multiple regression analyses indicated that accuracy was most strongly predicted by fluid intelligence and semantic understanding of emotion words in individuals with PSD, with significant additional effects of gender, history of substance use disorders in remission, and educational attainment. PSD group remained a significant predictor of accuracy after controlling for these factors. In summary, individuals with PSD and a history of interpersonal aggression exhibit more pronounced deficits in emotion recognition than those with PSD alone. This underscores the potential value of incorporating emotion recognition assessment and training into clinical interventions to reduce aggression risk and improve social functioning in individuals with PSD.

## Introduction

Emotion recognition is a fundamental component of human social interactions. The ability to recognize and correctly interpret non-verbal cues such as facial, vocal, or bodily expressions helps the individual to navigate in social situations. However, an impaired understanding of non-verbal cues of emotion may disrupt interactions and relationships^1^ and possibly contribute to inadequate responses such as interpersonal aggression. Interpersonal aggression in psychotic spectrum disorders (PSD), here defined as schizophrenia, schizoaffective disorder, delusional disorder, and bipolar disorder with psychotic features, is a major societal concern due to the human suffering caused by stigmatization^2,3^ and poor social functioning^4^ of individuals suffering from these disorders.

Multiple studies have identified impairments in both visual and auditory emotion recognition in individuals with schizophrenia spectrum disorders^5–12^ (for recent meta-analyses, see^13,14^). Most previous studies have focused on a limited number of basic emotions and have utilized static facial expressions^15^. However, the impairment in facial emotion recognition seems to occur also when individuals with schizophrenia are presented with dynamic stimuli, such as facial emotional expressions of varying intensity^16^ or human avatars^17^. In addition, impairments have also been reported for emotion recognition from body movements presented in form of point-light walkers^18,19^ or silhouettes^20^.

A systematic review indicated that deficits in emotion recognition were most pronounced for negative emotions, although difficulties were also evident in recognition of positive emotions in individuals with schizophrenia^7^. A recent study, using photographs of professional performers portraying seven different facial emotional expressions^21^, investigated misclassification patterns in these individuals. It highlighted issues such as lack of sensitivity to certain negative emotions, cross-valence misclassification between positive and negative emotional expressions (happiness misinterpreted as disgust), and within-valence misclassification of negative emotions (sadness classified as fear or anger). Even though the exact underlying mechanisms leading to such misclassifications might not yet be fully understood, a complex pattern of identified neurocognitive dysfunction, including disturbances in visual and auditory processing^22^ suggests a broader cognitive and perceptual impairment^23^.

Individuals with schizophrenia thus seem to experience difficulties not only with identification of emotional expressions but also with emotion valence accuracy, especially when decoding negative or neutral stimuli^24^. Previous research has reported greater tendencies to interpret neutral facial expressions as negative in individuals with this disorder as compared to healthy controls^25,26^. A link between emotion recognition, valence processing, and semantic understanding has been proposed^27^, suggesting difficulties stemming from semantic memory disorganization in individuals suffering from schizophrenia^28^. Deficits in the ability to correctly recognize and interpret emotions may also arise, in part, from reduced fluid and crystallized intelligence^29,30^, which are also linked to lower educational attainment^31^. In addition, greater severity of negative symptoms, such as apathy and flat affect^13^, has also been associated with reduced recognition accuracy. Effects such as these may potentially share underlying mechanisms, as emotion recognition has been shown to involve prefrontal regions associated with cognitive control, language, and working memory processes^32,33^.

Studies exploring emotion recognition ability specifically in individuals with schizophrenia spectrum disorders and a history of interpersonal aggression have provided mixed results regarding recognition of static facial expressions. Several studies indicated significantly worse overall performance on emotion recognition tasks in violent study participants as compared to their non-violent counterparts^34,35^, whereas others failed to confirm such differences^36,37^. Positive symptoms in schizophrenia have been connected to impairments for specific emotions, such as recognition of fear^36^, anger^37^, or sadness^38^ in violent individuals. In prior studies, including a subset of the current sample, we further observed that emotion recognition ability showed positive associations with psychomotor speed^39^ and negative associations with antipsychotic dosage^40^ in individuals with PSD and history of interpersonal aggression, potentially indicating confounding effects by higher antipsychotic doses on psychomotor speed and thus the ability to process and interpret temporally dynamic emotional expressions.

Given the paucity of studies examining individuals with a documented history of interpersonal aggression, such as physical violence or serious death threats, and need for more ecologically valid testing paradigms, this study investigates recognition of a wide range of both negative and positive emotions conveyed dynamically through facial, vocal, and bodily expressions^41^. More specifically, we assess emotion recognition in three modalities: visual (videos presented without sound), auditory (sound only), and multimodal (videos presented with sound). We compare (a) individuals with PSD and history of interpersonal aggression (PSD+AGG), (b) individuals with PSD but no prior history of interpersonal aggression (PSD-AGG), and (c) healthy community-based controls (HC). In addition to emotion recognition rates, we also investigate misclassification patterns in order to gain a complete picture of how emotion judgments vary across emotions and groups. Finally, we investigate relationships between emotion recognition ability and selected clinical, cognitive and sociodemographic variables, including cognitive ability as well as semantic understanding of emotion words.

We hypothesize misclassification of emotions to be greatest in PSD+AGG, intermediate in PSD-AGG, and lowest in HC. Based on the previous research, we expect misclassifications to occur across all presentation modalities (visual, auditory, and multimodal). We also expect performance on the emotion recognition task to be associated with semantic understanding of emotion words. In line with previous research, we anticipate both within- and cross-valence misclassifications to occur more frequently in the PSD groups as compared to HC.

## Methods

### Participants

This case control cross-sectional study contains three groups of participants (see Table 1): (a) PSD+AGG (*n* = 79) recruited at Forensic Psychiatry Stockholm; (b) PSD-AGG (*n* = 72) recruited at psychiatric outpatient clinics in Stockholm; and (c) HC (*n* = 86) recruited via the State Person Address Registry in Sweden. The study was conducted as part of the Stockholm Forensic Psychiatry Project, and a smaller subset of the current sample has been included in previous publications from this project^39,40,42^. Participant groups were matched by age and gender. All procedures were in accordance with the Swedish Research Council’s ethical guidelines and the Helsinki Declaration. Ethical approvals 2014/827-31/4, 2017/ 219-32, 2018/307-32, and 2019-01422 were provided by the Swedish Ethical Review Authority. All participants provided written informed consent.

**Table 1:** Clinical, cognitive and sociodemographic characteristics of the study participants.

	PSD+AGG	PSD-AGG	HC	Statistics
	*n* = 79 (a)	*n* = 72 (b)	*n* = 86 (c)	
Sex (male)	59 (74.7%)	50 (69.4%)	64 (74.4%)	0.663, *p* = 0.718^a^, a = b = c
Age (at consent)	36.46 (20–60)	39.10 (19–57)	35.06 (20–60)	5.744, *p* = 0.057^b^, a = b = c
Achieved education				
Swedish schooling (up to age of 16)	63 (79.7%)	65 (90.3%)	79 (91.9%)	6.271, *p* = 0.043^a^, all groups
				3.235, *p* = 0.072^a^, a = b
Primary school	41 (51.9%)	11 (15.2%)	3 (3.5%)	80.984, *p* < 0.001^a^, all groups
Secondary school	29 (36.7%)	43 (59.8%)	32 (37.2%)	22.754, *p* < 0.001^a^, a < b
Post-secondary education	9 (11.4%)	18 (25%)	51 (59.3%)	
Assessment				
WAIS: Matrix Reasoning (stanine scale 1–19)	7.72 (2–16)	9.35 (1–18)	12 (3–18)	51.779, *p* < 0.001^b^, all groups
				1970.5, *p* = 0.019^c^, a < b
				1773.5, *p* < 0.001^c^, b < c
Finger tapping dominant hand	60.91 (39–91)	63.32 (32–97)	72.72 (56–95)	53.806, *p* < 0.001^b^, all groups
				2378, *p* = 0.108^c^, a = b
Synonym test	12.26 (2–14)	12.19 (3–14)	12.94 (10–14)	1.323, *p* = 0.516^b^, a = b = c
Prior alcohol/substance use disorder				
Yes	60 (75.9%)	24 (33.3%)	6 (7%)	84.109, *p* < 0.001^a^, all groups
No	19 (24.1%)	48 (66.7%)	80 (93%)	27.716, *p* < 0.001^a^, a > b
Diagnosis and symptoms				
Schizophrenia	62 (78.5%)	62 (86.1%)	—	1.809, *p* = 0.405^a^, a = b
Schizoaffective disorder and other psychoses	10 (12.7%)	7 (9.7%)	—	
Bipolar disorder with psychosis	7 (8.9%)	3 (4.2%)	—	
Illness duration (years)	9.93 (0.5–31)	13.76 (0.8–37)	—	2213, *p* = 0.019^c^, a < b
SANS	23.49 (2–60)	22.22 (0–49)	2.59 (0–18)	134.901, *p* < 0.001^b^, all groups
				2352.5, *p* = 0.067^c^, a = b
SAPS	7.92 (0–53)	9.93 (0–79)	0.59 (0–14)	68.749, *p* < 0.001^b^, all groups
				2654.5, *p* = 0.471^c^, a = b
Medication				
Haloperidol mg/day	13.79 (0–70)	8.18 (0–36)	—	1792, *p* < 0.001^c^, a > b
Aggressive acts				
Threats to life	12 (15.2%)	—	—	
Assault	40 (50.6%)	—	—	
Grievous bodily harm	24 (30.4%)	—	—	
Murder/manslaughter	3 (3.8%)	—	—	

*SANS* the Scale for the Assessment of Negative Symptoms, *SAPS* the Scale for the Assessment of Positive Symptoms.

^a^Chi-square test.

^b^Kruskal–Wallis test.

^c^Mann–Whitney *U*-test.

Inclusion criteria for all participants included age between 20 and 60 years at the time of consent, and sufficient knowledge of the Swedish language to complete the study procedures. Prior substance abuse was allowed but needed to be in remission for at least 3 months prior to the testing. Inclusion criteria for the groups with PSD also incorporated the condition of clinical stability and no changes in medication for at least 3 months prior to the testing. Psychiatric comorbidities, including ADHD, autism spectrum disorders, mild intellectual disability, and personality disorders, were permitted. However, comorbidities of ADHD and autism spectrum disorders were excluded from statistical analyses due to the presence of only three participants with verified diagnoses made prior to prodromal phase or psychotic symptom onset.

Exclusion criteria for all participants included neurological disorders, untreated endocrine disorders, brain trauma prior to the onset of psychosis, active alcohol and/or substance abuse, moderate or severe intellectual disability, and diabetes type 1, a chronic disease that is known to cause mild to moderate decrements in cognitive functions due to cerebral microvascular changes^43^. Exclusion criteria for PSD-AGG included history of aggressive behavior evident from medical chart review, especially from admissions and self-reports. Exclusion criteria for the HC group also included any history of psychiatric disorders or a first- or second-degree relative with PSD.

### Clinical and cognitive assessment

All participants underwent a short semi-structured demographic interview and were asked about the presence of the studied psychiatric disorders in first- and second-degree relatives, prior interpersonal aggression, as well as prior alcohol and substance abuse. HC and PSD-AGG participants completed the self-rating scales Alcohol Use Disorders Identification Test (AUDIT)^44^ (Cronbach’s alpha = 0.80) and Drug Use Disorders Identification Test (DUDIT)^45^ (Cronbach’s alpha > 0.90). Participants who exceeded the cut-off (8 points on AUDIT and/or 6 points on DUDIT for males, 6 points on AUDIT and/or 2 points on DUDIT for females), or reported history of interpersonal aggression, were excluded from the study.

Participants were further screened for psychiatric disorders by a psychiatrist or a psychiatry resident, using Schedules for Clinical Assessment in Neuropsychiatry 2.1^46^. Current psychotic symptoms were rated using the Scale for the Assessment of Positive Symptoms^47^ and the Scale for the Assessment of Negative Symptoms^48^. Information about current antipsychotic medication was retrieved from patient medical files for the PSD groups, and doses were converted to haloperidol mg/day equivalents following Andreasen^49^.

Information about the type and number of aggressive acts in PSD+AGG group was obtained from available (a) crime reports from the Swedish Police Authority concerning criminal convictions, (b) forensic psychiatric assessments, (c) self-reports, and (d) hospital case reports prior to forensic psychiatric care. Computerized hospital case records from all public psychiatric care providers in the Stockholm area since 2007 were accessed. Aggressive acts, here defined as threats to life, assault, grievous bodily harm, or murder/manslaughter, were assessed following Cornell^50^. Participants with history of interpersonal aggression and index offence, such as arson/deliberate fire setting, robbery or theft were also included if interpersonal aggression had been observed and documented in hospital records or if they had prior convictions for physical acts of aggression. It was not possible to determine whether aggression occurred during substance use or withdrawal, either from participants’ interview responses or from police sentencing reports, because timely drug testing was often unavailable and would not have detected substances such as methylenedioxypyrovalerone, which was prevalent at the time.

Matrix Reasoning, a subtest of Wechsler Adult Intelligence Scale-IV (WAIS-IV)^51^, administered by psychologist, was used as a measure of fluid intelligence. A scaled score for every participant was obtained from the age-normed tables in the manual^52^.

Psychomotor speed was measured by a computerized finger tapping test, using Inquisit 5 software^53^. Participants were instructed to tap the spacebar with the index finger of the dominant hand in 5–10 test blocks, each lasting 10 s. The average score was calculated based on the number of finger taps.

### Emotion recognition tasks

Semantic understanding of emotion words was assessed by a synonym test developed by the research group. Participants were presented with 14 emotion words included in test protocols^40,41^ and were required to identify correct synonym for each emotion word in a multiple-choice task with three response options per item (e.g., for interest, the response options were sadness, enthusiasm, and irritation). Data from four participants (PSD+AGG, *n* = 1; PSD-AGG, *n* = 2; HC, *n* = 1) were considered unreliable due to procedural error (it was obvious that participants noticed emotion words presented on one side of the questionnaire only) and were thus excluded from further analyses.

After completing the synonym test, the Emotion Recognition Assessment in Multiple modalities test (ERAM)^41^ was used to assess emotion recognition. The ERAM test is based on stimuli from the Geneva Multimodal Emotion Portrayals corpus^54^, a database of five male and five female actors portraying different emotions using facial gestures, tone of voice, and body movement. It has previously been validated in a Swedish context using a large sample of healthy young adults (*N* = 593), showing good internal consistency and absence of ceiling or floor effects^41^. The vocal content is the same for all emotions and consists of pseudo-language sentences with no verbal meaning (e.g., “ne kali bam sud molen!”). The test contains 72 unique stimuli displaying 12 emotions representing all quadrants of the emotion circumplex^55^: positive valence and high arousal (happiness, pride), positive valence and low arousal (interest, pleasure, relief), negative valence and high arousal (anger, despair, disgust, fear), and negative valence and low arousal (anxiety, irritation, sadness). The inclusion of multiple emotions within each quadrant reduces the risk of inflated accuracy due to inference strategies based solely on valence or arousal. Stimuli were presented in three blocks: first 24 stimuli in a visual-only condition, followed by 24 stimuli in an auditory-only condition, and 24 stimuli in a multimodal (audio-visual) condition (2 stimuli per emotion and condition). Trials within blocks were presented in a fixed pseudo-randomized order.

After being presented with a stimulus, the participants were asked to identify a target emotion in a forced-choice paradigm. The alternatives consisted of the 12 target emotions and appeared on the screen after every stimulus presentation. Participants were instructed to respond spontaneously and without overthinking, although no explicit time limits were imposed. Duration of each presentation was between 1 and 5 s. The stimuli were presented on 23” LED-screen (Dell, E2314Hf). The sound levels were normalized and sound played on Dell AY410 Multimedia Speaker System. Distance between the screen and participants was between 50 and 70 cm. Test duration was approximately 15–20 min.

### Statistical analyses

Descriptive statistics were used to analyze the data obtained through clinical and cognitive assessment. Normality was assessed both visually and using the Shapiro-Wilk test. To analyze continuous variables, such as age, fluid intelligence, psychomotor speed, semantic understanding of emotion words, illness duration, positive and negative symptoms, and antipsychotic dose/day, Kruskal–Wallis and Mann–Whitney *U* tests were conducted to compare all three groups or two groups, respectively. Categorical variables, such as gender, education, prior alcohol/substance use disorder, and diagnosis, were analyzed by Chi-square tests.

To gain insights into participants’ ability to recognize emotions, four confusion matrices based on the raw data were created. Confusion matrices plotted target emotions in the rows and percentage of selected emotions in the columns, separately for each emotion and participant group, both for overall accuracy and accuracy in different modalities. Confidence intervals based on the mean values for every participant were calculated.

An index was created for *within-valence* (positive target emotions misclassified as other positive emotions, negative target emotions misclassified as other negative emotions) and *cross-valence misclassifications* (positive target emotions misclassified as negative emotions, negative target emotions misclassified as positive emotions) in all three participant groups. Chi-square analyses were conducted to compare the differences in within- and cross-valence misclassifications between the groups.

Unbiased hit rates (Hu) were calculated to correct for systematic response biases which may otherwise artificially inflate accuracy in forced-choice tasks^56^. For example, a participant could appear to achieve 100% accuracy for anger by labeling all stimuli as angry, despite having no ability to distinguish anger from other emotions. Hu, ranging from 0 to 1, was calculated using following formula: *hit rate* × *(1 - false alarms)*. A maximum score of 1 would indicate that all stimuli of a particular emotion were classified correctly and that the corresponding response was never used for other emotions. This correction resembles signal detection methods; however, unlike standard signal detection terms, it permits separate analyses across individual stimulus categories. Due to small number of items per emotion and modality, Hu was not calculated for individual emotions separately for each presentation modality. Therefore, the Hu scores for each participant and (a) each modality (summing the emotions), as well as (b) each emotion (across all modalities), were calculated.

Three separate mixed analyses of variance (ANOVA) were conducted, using Hu scores as the dependent variable. All three ANOVAs included group (three levels: PSD+AGG, PSD-AGG, HC) as a between-subjects variable. The first ANOVA further examined effects of presentation modality (within-subjects variable with 3 levels: visual, auditory, multimodal) on emotion recognition across all emotions in a 3 (group) × 3 (modality) design. The second ANOVA instead examined effects of emotion (within-subjects variable with 12 levels: pride, irritation, happiness, anger, pleasure, disgust, interest, anxiety, relief, fear, sadness, despair) on emotion recognition in a 3 (group) × 12 (emotion) design. The third ANOVA examined effects of valence (within-subjects variable with two levels: positive, negative) and arousal (within-subjects variable with two levels: high, low) on emotion recognition in a 3 (group) × 2 (valence) × 2 (arousal) design. *Positive emotions* included pride, happiness, pleasure, interest, and relief. *Negative emotions* included irritation, anger, disgust, anxiety, fear, sadness, and despair. *High arousal emotions* included pride, happiness, anger, disgust, fear, and despair. *Low arousal emotions* included irritation, pleasure, interest, anxiety, relief, and sadness. In addition, we repeated all ANOVAs, including gender (female, male) as an additional between-subjects factor to test whether it systematically interacted with group effects, given evidence that gender influences emotion recognition, with females often outperforming males^41,57,58^.

Bivariate correlation analyses (Pearson *r* and Spearman *ρ*) were conducted to examine relationships between overall emotion recognition accuracy (Hu) and relevant clinical, cognitive, and sociodemographic variables (see Table 1) in individuals with PSD. A hierarchical multiple regression was further conducted to examine how well overall emotion recognition accuracy (Hu) could be predicted by the background variables in individuals with PSD, and what variables would emerge as important predictors. The following predictors were entered simultaneously in the first step: semantic understanding of emotion words, negative and positive symptoms of psychosis, fluid intelligence, psychomotor speed, antipsychotic dose/day, illness duration, prior alcohol/substance use, education, and gender. In the second step, group (PSD+AGG, PSD-AGG) was entered in order to examine whether it would be a significant predictor of emotion recognition accuracy when controlling for the other background variables. Listwise exclusion was applied for missing variables.

All statistical analyses were conducted in SPSS version 29.0.2.0.

## Results

### Clinical, cognitive, and sociodemographic characteristics

As shown in Table 1, more than two-thirds of participants were males, though it should be noted that the gender composition was matched across groups. More than a half of participants in the PSD+AGG group had not completed post-primary education as compared to 15.3% in PSD-AGG group. The HC group had the highest level of education, with almost 60% having completed post-secondary education. The scores on Matrix Reasoning (WAIS-IV) indicated a significant difference between PSD+AGG and PSD-AGG groups, with average scores of 7.72 and 9.35, respectively, on a stanine scale 1–19. There was a significant difference between HC and PSD groups on the finger tapping test measuring psychomotor speed, but no significant difference between PSD+AGG and PSD-AGG. No significant difference in performance on the synonym test was observed amongst the groups. Prior alcohol and/or substance abuse were most prominent in the PSD+AGG participant group, reaching 75.9%, as compared to 33.3% in PSD-AGG and 7% in HC group. A majority of participants in the PSD+AGG and PSD-AGG groups had schizophrenia as their primary diagnosis, 78.5% and 86.1%, respectively, and were treated with antipsychotic medication. There was no significant difference in negative and positive symptom severity between PSD groups, although illness duration was significantly longer in PSD-AGG (13.76 years) compared to PSD+AGG (9.93 years). Approximately half of the participants in PSD+AGG group had committed assault. A third of the participants from the same group were convicted of grievous bodily harm. Aggressive acts such as threats to life and murder/manslaughter were represented by approximately 15% and 3.8%, respectively.

### Misclassification analyses

Table 2 presents the confusion matrix across all modalities for each participant group. The values presented in main diagonals indicate the percentage of correct recognition. The values in other cells indicate the percentage of incorrect answers in the corresponding emotion category. Accuracy was above chance level (calculated as (1/12) × 100 = 8.33%) for all emotions for each participant group, as indicated by 95% confidence intervals that did not overlap with the chance-level. For reference, overall accuracy for the HC group (53%) was 6.3 times higher than chance-level performance, which is similar to the norms for the ERAM test presented in the original validation study (56%)^41^.

**Table 2:**
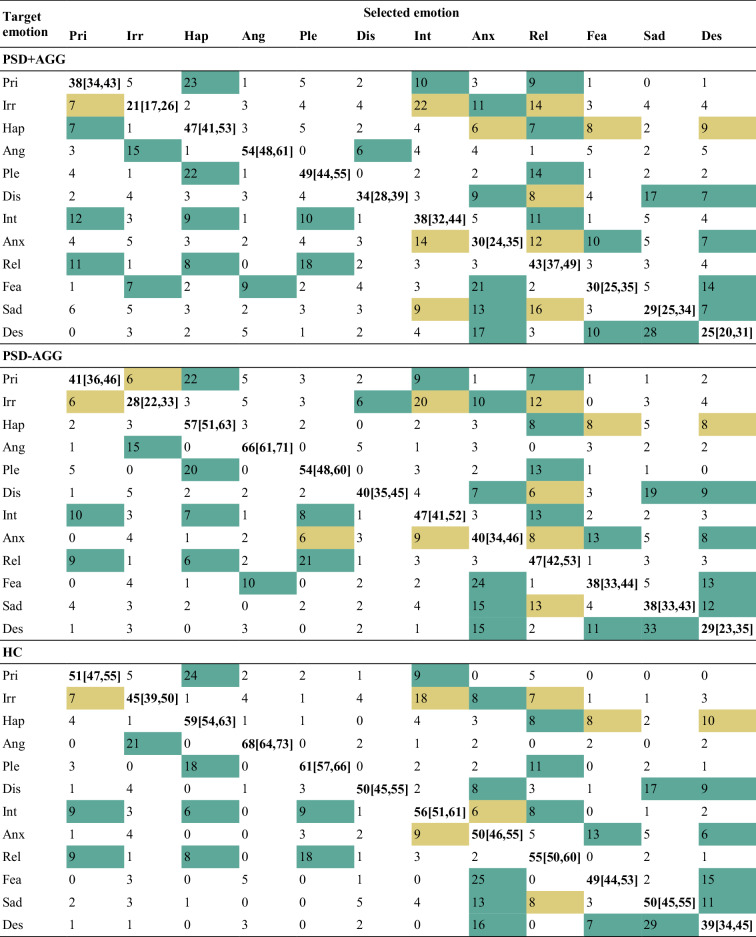
Confusion matrix illustrating overall accuracy across all modalities in the ERAM test for all three groups.

Diagonals marked in bold represent percentages of correctly identified emotions: Pri (pride), Irr (irritation), Hap (happiness) Ang (anger), Ple (pleasure), Dis (disgust), Int (interest), Anx (anxiety), Rel (relief), Fea (fear), Sad (sadness), and Des (despair). Values in brackets indicate 95% CIs. Green cells represent within-valence misclassifications with frequency above 5%. Yellow cells represent cross-valence misclassifications with frequency above 5%.

Below, we summarize within- and cross-valence misclassifications that occurred with a frequency above 5% across all modalities, as shown in Table 2. Confusion matrices are also shown separately for each modality (visual, auditory, and multimodal) and group in Supplementary Tables S1, S2, and S3.

#### Positive target emotions misclassified as other positive emotions

All three participant groups misclassified (a) pride as happiness and interest, (b) happiness as relief, (c) pleasure as happiness and relief, (d) interest as pride, happiness, pleasure, and relief, and (e) relief as pride, happiness, and pleasure. Happiness was also misclassified as pride in PSD+AGG, and pride as relief in both PSD groups.

#### Positive target emotions misclassified as negative emotions

Happiness was mistaken for fear and despair by all participant groups. PSD+AGG also misclassified happiness as anxiety, PSD-AGG misclassified pride as irritation, and HC at times mistook interest for anxiety.

#### Negative target emotions misclassified as other negative emotions

All three groups misclassified (a) irritation as anxiety, (b) anger as irritation, (c) disgust as anxiety, sadness, and despair, (d) anxiety as fear and despair, (e) fear as anxiety and despair, (f) sadness as anxiety and despair, and g) despair as anxiety, fear, and sadness. PSD+AGG mistook anger for disgust and fear for irritation. PSD-AGG misclassified irritation as disgust. Fear was more commonly misclassified as anger by both PSD groups.

#### Negative target emotions misclassified as positive emotions

All three groups misclassified (a) irritation as pride, interest, and relief, (b) anxiety as interest, and (c) sadness as relief. Furthermore, both PSD groups mistook disgust and anxiety for relief. PSD+AGG also misclassified sadness as interest, whilst PSD-AGG misclassified anxiety as pleasure.

#### Misclassification index

Table 3 presents within- and cross-valence misclassifications for all three participant groups across all modalities. Most misclassifications occurred between conceptually similar emotions of the same valence for all three participant groups. Within-valence misclassifications of negative emotions were further the most common regardless of the group. However, frequency of both cross- and within-valence misclassifications of positive emotions was, in general, more prominent in PSD groups than in the HC group.

**Table 3:** Misclassification index illustrating percentage of misclassifications for positive and negative emotions for every participant group.

Target emotions	Misclassified as	PSD + AGG *n* = 79(a)	PSD-AGG *n* = 72 (b)	HC *n* = 86 (c)	Statistics (Chi^2^)
Positive	Positive (other)	16.14 [16.01, 16.27]	14.54 [14.42, 14.67]	13.42 [13.32, 13.52]	5.3, *p* = 0.021* a > b 17.45, *p* < 0.001* a > c 2.97, *p* = 0.085 b/c, ns
	Negative	7.59 [7.42, 7.77]	6.62 [6.48, 6.75]	4.76 [4.71, 4.82]	3.92, *p* = 0.048* a > b 41.35, *p* < 0.001* a > c 18.29, *p* < 0.001* b > c
Negative	Negative (other)	25.18 [25.01, 25.34]	25.15 [25.00, 25.31]	22.06 [21.92, 22.20]	0.002, *p* = 0.97 a/b, ns 15.98, *p* < 0.001* a > c 15.03, *p* < 0.001* b > c
	Positive	14.52 [14.34, 14,70]	9.92 [9.78, 10.05]	6.93 [6.83, 7.03]	53.26, *p* < 0.001* a > b 180.93, *p* < 0.001* a > c 33.11, *p* < 0.001* b > c

^*^Statistically significant *p* < 0.05

Values in brackets indicate 95% CIs. Comparisons of misclassifications based on Chi-square test.

Chi-square analyses comparing PSD+AGG and PSD-AGG indicated significantly higher severity of misclassification in the PSD+AGG group both in misclassifying negative target emotions as positive emotions (*X*^2^ = 53.26, *p* < 0.001), positive target emotions as other positive emotions (*X*^2^ = 5.3, *p* = 0.021), and positive target emotions as negative emotions (*X*^2^ = 3.92, *p* = 0.048). The difference in misclassifying negative target emotions as other negative emotions was not significant (*X*^2^ = 0.002, *p* = 0.979) between PSD groups.

### Unbiased hit rate as a function of group and modality, emotion, and valence/arousal

#### Group effects

**Fig. 1:**
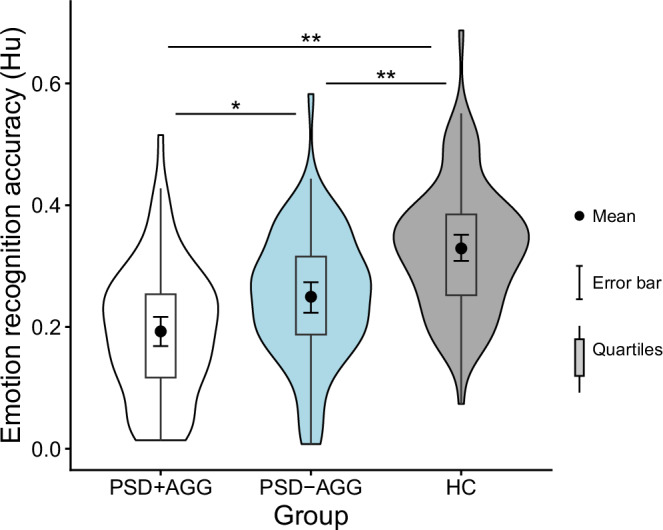
Violin plots based on unbiased hit rates (Hu) illustrating the distribution of overall accuracy in the ERAM test in all participant groups. *Note*. Error bars indicate 95% confidence intervals. *p = 0.001, **p < 0.001.

The first ANOVA yielded a significant main effect of *group F*(2, 234) = 35.64, *p* < 0.001, *η*_*p*_^2^ = 0.23, suggesting that accuracy of emotion recognition in HC (*M* = 0.33, SD = 0.11) was significantly higher than in PSD+AGG (*M* = 0.19, SD = 0.11), and PSD-AGG (*M* = 0.25, SD = 0.11), as shown in Fig. 1. Independent *t*-tests (two-tailed) confirmed statistically significant differences between (a) PSD+AGG and PSD-AGG: *t*(149) = −3.26, *p* = 0.001, 95% *CI* [−0.09, −0.02], (b) PSD+AGG and HC: *t*(163) = −8.22, *p* < 0.001, 95% *CI* [−0.17, −0.10], as well as (c) PSD-AGG and HC: *t*(156) = −4.70, *p* < 0.001, 95% *CI* [−0.11, −0.05]. By design, the group effects in the second and third ANOVA (see below) were identical to the one shown in Fig. 1. Additional group effects are therefore not reported below, in order to avoid redundancy.

#### Modality effects

The main effect of *modality* in the first ANOVA was significant *F*(1.955, 457.565) = 114.29, *p* < 0.001, *η*_*p*_^2^ = 0.33. Pairwise comparisons suggested that emotion recognition accuracy in multimodal presentation (M = 0.37, SD = 0.17) was significantly higher than recognition in visual (*p* < 0.001) or auditory (*p* < 0.001) modality, and that emotion recognition in the visual modality (*M* = 0.27, SD = 0.13) was significantly higher (*p* < 0.001) than in the auditory modality (*M* = 0.25, SD = 0.14). No significant interaction effect between group and modality was observed *F*(3.911, 457.565) = 1.88, *p* < 0.114, *η*_*p*_^2^ = 0.02. Mauchly’s test indicated that the assumption of sphericity had been violated, *W* = 0.96, *X*^2^(2) = 9.49, *p* = 0.009, and therefore degrees of freedom were corrected using Huynh-Feldt estimates of sphericity (*ε* = 0.978).

#### Emotion effects

The second ANOVA yielded a significant main effect of *emotion*, *F*(9.78, 2289.17) = 87.81, *p* < 0.001, *η*_*p*_^2^ = 0.27. In general, anger (*M* = 0.50, SD = 0.26), pleasure (*M* = 0.35, SD = 0.21), disgust (M = 0.32, SD = 0.23) and happiness (*M* = 0.28, SD = 0.17) were the best recognized emotions whereas anxiety (*M* = 0.16, SD = 0.15), despair (*M* = 0.16, SD = 0.17), irritation (M = 0.17, SD = 0.18) and sadness (*M* = 0.19, SD = 0.17) had lower accuracy. No significant interaction effect between group and emotion was observed *F*(19.57, 2289.17) = 1.002, *p* < 0.456, *η*_*p*_^2^ = 0.008. Supplementary Table S4 presents descriptive statistics and pairwise comparisons with Holm-Bonferroni correction for all emotions across all three participant groups. Mauchly’s test indicated that the assumption of sphericity was not met, *W* = 0.38, *X*^2^(65) = 221.85, *p* < 0.001, so degrees of freedom were Huynh-Feldt corrected (*ε* = 0.889).

#### Effects of valence and arousal

The third ANOVA yielded a significant main effect of *valence F*(1, 234) = 21.03, *p* < 0.001, *η*_*p*_^2^ = 0.08, indicating that emotions with positive valence (*M* = 0.28, SD = 0.13) were slightly better recognized than emotions with negative valence (*M* = 0.25, SD = 0.13). The main effect of *arousal* was also significant *F*(1, 234) = 149.89, *p* < 0.001, *η*_*p*_^2^ = 0.39, suggesting better recognition of high-arousal emotions (*M* = 0.29, SD = 0.13) than low-arousal emotions (*M* = 0.23, SD = 0.13). The main effects were qualified by a significant valence × arousal interaction *F*(1, 234) = 11.49, *p* < 0.001, *η*_*p*_^2^ = 0.05, reflecting that low-arousal negative emotions (anxiety, irritation, sadness) showed the lowest recognition rate. No interaction effect between group and valence *F*(2, 234) = 1.13, *p* = 0.324, *η*_*p*_^2^ = 0.01, group and arousal *F*(2, 234) = 1.90, *p* = 0.152, *η*_*p*_^2^ = 0.016, or group, valence and arousal was observed *F*(2, 234) = 0.12, *p* = 0.886, *η*_*p*_^2^ = 0.001.

#### Supplementary ANOVAs

Additional analyses, including gender as a between-subjects factor, replicated findings from the main analyses regarding effects of group, modality, emotion, valence, and arousal, see Supplementary Table S5. No significant main effects of gender or interaction effects between group and gender were observed.

### Associations between emotion recognition accuracy and clinical, cognitive, and sociodemographic variables in the PSD groups

Bivariate correlations (both Pearson *r* and Spearman *ρ*) between overall emotion recognition accuracy (Hu) and background variables are presented in Supplementary Table S6. Emotion recognition accuracy was positively correlated with group (0 = PSD+AGG, 1 = PSD-AGG) (*r* = 0.26, *p* < 0.001), confirming better emotion recognition ability for PSD-AGG vs. PSD+AGG. In addition, higher emotion recognition was associated with higher education (*r* = 0.30, *p* < 0.001), fluid intelligence (*r* = 0.55, *p* < 0.001), psychomotor speed (*r* = 0.28, *p* < 0.001), and semantic understanding of emotion words (*ρ* = 0.46, *p* < 0.001). Negative associations were instead observed between emotion recognition and antipsychotic dose/day (*ρ* = −0.21, *p* = 0.009) and negative symptoms (*r* = −0.25, *p* = 0.002). Overall, the background variables showed only small to medium intercorrelations, making them suitable as predictors in a multiple regression analysis.

A hierarchical multiple regression analysis (tolerance > 0.6, variance inflation factor <1.6) was performed in two steps (see Table 4). This analysis included the PSD groups only (*N* = 137, due to missing data for 14 participants for at least one of the included variables). In the first step all clinical, cognitive, and sociodemographic variables were entered simultaneously, and the model explained 41.4% of variance in emotion recognition accuracy, *R*^2^_adj_ = 0.414, *F*(10, 126) = 10.61, *p* < 0.001. In the second step, group (PSD+AGG, PSD-AGG) was entered, explaining an additional 2.1% of variance in emotion recognition, Δ*R*^2^ = 0.021, Δ*F*(1, 125) = 4.98, *p* = 0.027, with final *R*^2^_adj_ = 0.432. Fluid intelligence (*β* = 0.364, *p* < 0.001), semantic understanding of emotion words (*β* = 0.286, *p* < 0.001), gender (*β* = 0.176, *p* = 0.014), and education (*β* = 0.156, *p* = 0.035) were significant predictors of emotion recognition in the final model, consistent with their effects in the first step. In addition, group (*β* = 0.177, *p* = 0.027) and prior alcohol or substance abuse (*β* = 0.163, *p* = 0.037) emerged as significant predictors of emotion recognition accuracy in the final model.

**Table 4:** Hierarchical regression analysis predicting overall emotion recognition accuracy (Hu) in individuals with PSD: Effects of clinical, cognitive and sociodemographic variables (step 1) and group (PSD+AGG, PSD-AGG) (step 2).

Variable	*B*	95% CI		SE	*β*	*R*^2^	Adj. *R*^2^	ΔR^2^	ΔF	*p*
		LL	UL							
Model 1						0.46	0.414	0.457	10.61	<0.001
Constant	−0.072	−0.191	0.047	0.060						0.232
Illness duration	−0.001	−0.003	0.001	0.001	−0.067					0.341
Antipsychotic dose/day	−0.001	−0.002	0.001	0.001	−0.081					0.274
Education^a^	0.045	0.012	0.078	0.017	**0.196**					0.008
Fluid intelligence	0.011	0.007	0.016	0.002	**0.388**					<0.001
SANS	0.000	−0.002	0.001	0.001	−0.047					0.502
SAPS	0.000	−0.001	0.001	0.001	−0.008					0.904
AUD/SUD^b^	0.024	−0.009	0.056	0.016	0.107					0.150
Psychomotor speed	0.001	−0.001	0.002	0.001	0.056					0.450
Synonym test	0.011	0.005	0.018	0.003	**0.262**					<0.001
Gender^c^	0.045	0.010	0.080	0.018	**0.181**					0.035
Model 2						0.48	0.432	0.021	40.98	0.027
Constant	−0.132	−0.206	−0.003	0.065						0.044
Illness duration	−0.001	−0.003	0.001	0.001	−0.090					0.201
Antipsychotic dose/day	−0.001	−0.002	0.001	0.001	−0.059					0.418
Education^a^	0.036	0.003	0.070	0.017	**0.156**					0.035
Fluid intelligence	0.010	0.006	0.015	0.002	**0.364**					<0.001
SANS	0.000	−0.001	0.001	0.001	−0.033					0.634
SAPS	0.000	−0.001	0.001	0.001	−0.026					0.709
AUD/SUD^b^	0.036	0.002	0.070	0.017	**0.163**					0.037
Psychomotor speed	0.000	−0.001	0.002	0.001	0.046					0.530
Synonym test	0.012	0.006	0.019	0.003	**0.286**					<0.001
Gender^c^	0.043	0.009	0.078	0.017	**0.176**					0.014
Group^d^	0.039	0.004	0.074	0.018	**0.177**					0.027

Significant predictors are marked in bold. *N* = 137.

*CI* confidence interval, *LL* lower limit, *UL* upper limit, *SANS* Scale for the Assessment of Negative Symptoms, *SAPS* Scale for the Assessment of Positive Symptoms, *AUD* alcohol use disorder, *SUD* substance use disorder.

^a^0 = compulsory education only, 1 = higher than compulsory education.

^b^0 = no prior AUD/SUD, 1 = prior AUD/SUD.

^c^0 = male, 1 = female.

^d^0 = PSD+AGG, 1 = PSD−AGG.

## Discussion

This study investigated two complementary indices of emotion processing – emotion recognition accuracy and misclassification patterns—in individuals with PSD and a history of documented interpersonal aggression, a group often excluded from research due to comorbidity and illness severity. By testing participants during a phase of clinical stability, we minimized the influence of state effects, as well as acute effects of intoxicants. Emotion judgements were examined for a broad range of visual, auditory, and multimodal expressions featuring five positive and seven negative emotions. Across analyses, the PSD+AGG group demonstrated significantly lower emotion recognition accuracy than the PSD-AGG group, whose impairments were, in turn, significantly greater than those in the HC group. Misclassification patterns were largely consistent across presentation modalities, and the most distinctive finding was a markedly higher rate of cross-valence misclassifications in the PSD+AGG group, compared to the PSD-AGG and HC groups, particularly for negative emotions being misinterpreted as positive. Both PSD groups also showed more within-valence misclassifications than HC, and the PSD+AGG group misclassified positive emotions as other positive emotions significantly more frequently than the PSD-AGG group. Finally, emotion recognition accuracy in the PSD groups was significantly predicted by fluid intelligence, semantic understanding of emotion words, group, gender, prior alcohol or substance abuse, and educational attainment.

Overall, we replicated findings from prior studies showing reduced recognition of basic emotions (i.e., anger, disgust, fear, happiness, sadness) from visual^14^ and auditory^13^ stimuli in PSD groups as compared to HC. Furthermore, we observed reduced recognition in PSD also for multimodal expressions and additional emotions (i.e., anxiety, despair, interest, irritation, pleasure, pride, and relief) that have so far received less attention. While prior studies have often emphasized deficits in recognition of negative emotions in PSD^59^, we found similar deficits across both positive and negative emotions. It should be noted that previous studies have included a limited selection of positive emotions, in many cases only happiness, and thereby had a limited ability to examine misclassification patterns across emotional valence. Our results suggest that including a broader selection of both positive and negative emotions enables a more sensitive assessment of emotion recognition deficits in PSD. We observed large group effects, but no interaction effects in our analyses, which suggests that deficits in accuracy were similar across conditions. This could suggest that deficits may originate in general cognitive and perceptual impairments rather than emotion or modality specific impairments^22,23^.

Our finding that the PSD+AGG group showed consistently lower emotion recognition accuracy than the PSD-AGG group is further in line with previous studies that used facial expression stimuli^34,35,39,60^. It should, however, be noted that some previous studies have also reported no significant decline in emotion recognition for violent compared to non-violent PSD groups^36,37^. Krakowski et al.^36^ further reported that impaired emotion recognition was similarly associated with violent behavior in individuals with schizophrenia and non-psychotic violent individuals. A recent study also reported no association between emotion recognition and lifetime aggression^61^. Such discrepancies might be explained by differences regarding the design of emotion recognition tasks and the definition of aggressive behavior—which could have dissipated the association we identified between documented history of interpersonal aggression and severity of emotion recognition impairments. Nevertheless, the evidence for linking deficits in emotion recognition to aggression remains inconclusive.

Turning next to misclassification patterns, detailed analyses revealed that most misclassifications occurred between conceptually similar emotions of the same valence regardless of participant group. These findings are similar to the ones obtained in the validation study of the ERAM test in a community sample^41^ where within-valence misclassifications, such as anxiety being mistaken for fear, despair for sadness, sadness for fear, and pride for happiness, were reported as the most common. It is noteworthy that whilst both within- and cross-valence misclassifications occurred more frequently in the PSD groups than in the HC group, the PSD groups did not differ in prevalence of misclassifying negative target emotions as other negative emotions. Previous studies of facial recognition of basic emotions in individuals with schizophrenia have reported misclassifications among anger, sadness, and fear^21^ and linked those errors to severity^62^ and stage of illness^63^. It remains a possibility that the limited set of response options in forced-choice tests might have systematically inflated these misclassifications, especially if the other response options were happiness, disgust, and surprise—which are emotions with profoundly distinct visual representations^64^. Differences in findings may therefore reflect variations in methods and paradigms, as well as sample characteristics such as fluid intelligence and illness stage and severity.

Fluid intelligence, semantic understanding of emotion words, and educational level emerged as significant predictors of emotion recognition accuracy in PSD in the multiple regression analysis, which is in line with previous meta-analytic findings^29^ and our previous studies based on a subset of the current cohort^39,40^. These associations may reflect shared mechanisms, as explicit emotion recognition also involves prefrontal systems that support executive processes and language^32^. Moreover, individuals with PSD and a history of aggression show more pronounced deficits in top-down cognitive control, highlighting the role of executive dysfunction in this group^65^. However, in contrast with our previous studies^39,40^, we found no evidence for a significant role of psychomotor speed for emotion recognition in the current, larger sample when controlling for the other background variables. We also did not observe any significant associations between symptom severity and emotion recognition performance in the multiple regression analysis, which contrasts with a recent meta-analysis^13^ that identified negative symptoms as a strong predictor of emotion recognition deficits. It is possible that the choice of assessment instruments and analysis methods may underlie this difference.

Gender also emerged as a significant predictor of emotion recognition in the multiple regression analysis, indicating that females outperformed males, which is in line with previous studies^41,57,58^. However, gender effects did not interfere with our demonstration of group effects in emotion recognition, as supplementary ANOVAs showed no interaction effects between group and gender. Prior substance abuse further significantly predicted higher emotion recognition performance in the multiple regression analysis. This contrasts with previous research, which has linked substance abuse with lower social cognitive skills^66,67^. We believe this finding should be interpreted with caution for several reasons. First, participants in the current study had been in remission for at least 3 months prior to testing, which may have attenuated effects of substance use and withdrawal^68^. Second, our dichotomous variable did not capture important aspects of prior substance use, such as cumulative burden, severity, duration, or polysubstance use^69^. Third, although model assumptions were met, the shift in the association between substance abuse and emotion recognition from a non-significant negative bivariate correlation to a significant positive regression coefficient suggests a possible suppression effect. Closer inspection revealed that the coefficient changed sign when education, fluid intelligence, and group were added to the model. Because the regression coefficient reflects the unique variance in substance abuse after shared variance with these variables has been removed, and because these variables were both related to emotion recognition and differed across PSD groups, the substantive meaning of the positive regression coefficient remains unclear. In general, potential interactions among substance abuse, education, fluid intelligence, and group complicate interpretation of their relative contributions to emotion recognition accuracy and warrant further research. Nevertheless, group remained a significant predictor of emotion recognition accuracy even after controlling for clinical, cognitive, and sociodemographic factors. Because history of aggression was the primary distinction between the PSD groups, this result suggests that aggression may have contributed to the observed differences in emotion recognition, although the influence of other unmeasured group differences cannot be ruled out.

Our study yielded an unexpected clinically significant finding regarding misclassification of negative target emotions as positive emotions. This kind of misclassification occurred in 14% of cases in the PSD+AGG group, which was 1.2 times as common as in the PSD-AGG group, and twice as common as in the HC group. Because we are not aware of any prior studies examining such a broad set of positive emotions, direct comparison of our findings with existing literature is not currently possible. Nevertheless, if the above figure of 14% is reproducible, it could possibly have real-life implications for social interactions. We speculate that individuals in the PSD+AGG group may underweight negative social cues and/or overemphasize approach-oriented expectations. Such biases in prediction^70,71^ could produce negative-to-positive misclassifications, leading individuals to misinterpret signals that their behavior causes negative emotions in others^72^. This could, in turn, increase the likelihood that they fail to receive a social stop signal for their behavior^73^ and therefore adopt offensive strategies rather than non-aggressive ones such as withdrawal.

### Limitations and future directions

Several limitations need to be acknowledged. First, the cross-sectional design limits causal interference. Future studies could employ longitudinal designs to investigate if emotion recognition ability and other factors, such as childhood trauma^61^, contribute causally to interpersonal aggression in PSD. Second, we examined potential vulnerabilities to aggression rather than directly examining emotion recognition in individuals who are feeling agitated, irritable, or suffering from acute psychosis. Hence, there may be further state effects that compound the risk of aggression that were not possible to address in the current study.

Several caveats further relate to the design and administration of the emotion recognition tasks. First, the synonym test was developed specifically for this project and has not been validated at a population level, as it was not intended to serve as a screening tool for broad emotion-word comprehension. Rather, it was developed for use with the specific words employed in the emotion recognition tasks^40,41^ used in the current project. Second, the ERAM test relies on actor portrayals, which may differ in subtle but systematic ways from spontaneous expressions^74,75^. In addition, the sample was heterogenous with respect to ethnic background, which may have influenced performance on the test using stimuli depicting Caucasian European men and women. The stimuli were developed in Switzerland and may include paralinguistic features, such as intonation patterns, that are less common in Scandinavia, potentially affecting interpretation. Moreover, the ERAM test was administered last in the assessment battery, which may have reduced data quality in healthy individuals and those in the PSD-AGG group. Yet this would have biased the results in favor of the PSD+AGG group, who did the task on a separate day to interviews but nevertheless showed the lowest accuracy. Additional limitations include the limited number of stimuli per emotion, which may affect the stability of the estimates for individual emotions, as well as the relatively large number of response options, which may increase cognitive load. Finally, test performance likely reflects not only emotion recognition ability but also broader cognitive factors^30^, making these processes difficult to fully disentangle. Further studies could investigate potential group differences in gaze patterns^76^ or neural activation^23,77,78^ during emotion recognition to provide additional insights.

Clinically, our findings support the potential value of assessing emotion recognition in early stages of forensic and general psychiatric care. Given initial evidence that emotion recognition can be improved through targeted training in individuals with PSD^79,80^, future studies could examine whether such gains translate into improved social functioning and reduced aggression risk. Moreover, these findings have clinical implications for how emotions are communicated to individuals with PSD by family members and professionals. One possible recommendation is to intentionally combine facial expressions, paralinguistic cues, and clear verbal communication when conveying emotions in interactions with these individuals in order to support more accurate interpretation.

## Conclusion

In conclusion, this study examined emotion recognition in individuals with PSD, both with and without a history of interpersonal aggression. To the best of our knowledge, this is the first study examining five positive emotions in PSD. We discovered that individuals with PSD and history of interpersonal aggression exhibited more pronounced impairments in emotion recognition than those with PSD only. This effect remained significant after controlling for fluid intelligence, semantic understanding of emotion words, gender, history of substance abuse in remission, and educational attainment, all of which also predicted recognition accuracy. Our findings also suggest that misclassification of negative target emotions as positive emotions could be higher in individuals with PSD and history of interpersonal aggression than in their counterparts without such history.

## Supplementary information

Supplementary materials

## Data Availability

The data that support the findings of this study are available on request from the corresponding author [G.G.]. The data are not publicly available due to European regulations to protect participant privacy from the sharing of sensitive health data at an individual level.
