# Supplementary material for: Emotion recognition misclassification patterns in individuals with psychotic spectrum disorders and history of interpersonal aggression
Source: Schizophrenia (Heidelb). 2026 Jun 24;12(1):55. doi: 10.1038/s41537-026-00776-5 (PMC13294495; doi:10.1038/s41537-026-00776-5)
Supplement: Supplementary file 1 — Supplementary materials [file 41537_2026_776_MOESM1_ESM.docx]

**Supplementary materials for:**

**Emotion recognition misclassification patterns in individuals with psychotic spectrum disorders and history of interpersonal aggression**

Gabriela Gavalova^1,2,3*^, Petri Laukka^4^, Lennart Högman^5^, Malin V. Källman^1,3^, Marianne Kristiansson^1,3^, Märta Wallinius^6,7,8^, Håkan Fischer^5,9,10,11^ & Anette G. M. Johansson^1,3,12^

^1^Department of Clinical Neuroscience, Karolinska Institutet, Stockholm, Sweden

^2^Forensic Psychiatry Unit, Uppsala University Hospital, Uppsala, Sweden

^3^Centre for Psychiatry Research, Karolinska Institutet, Stockholm, Sweden

^4^Department of Psychology, Uppsala University, Uppsala, Sweden

^5^Department of Psychology, Stockholm University, Stockholm, Sweden

^6^Evidence-Based Forensic Psychiatry, Department of Clinical Sciences Lund, Psychiatry, Lund University, Lund, Sweden

^7^Center of Ethics, Law and Mental Health, Institute of Neuroscience and Physiology, The Sahlgrenska Academy, University of Gothenburg, Gothenburg, Sweden

^8^Research Department, Regional Forensic Psychiatric Clinic, Växjö, Sweden

^9^Aging Research Center, Department of Neurobiology, Care Sciences and Society, Karolinska Institutet, Sweden

^10^Department of Behavioural Sciences and Learning, Division of Psychology, Linköping University

^11^Stockholm University Brain Imaging Centre (SUBIC), Stockholm University, Sweden

^12^Ramsay Clinic Caloundra, Caloundra, Qld, Australia

| **Table S1.** Confusion matrix illustrating accuracy in visual modality in the ERAM test for all three groups. | | | | | | | | | | | | |
| --- | --- | --- | --- | --- | --- | --- | --- | --- | --- | --- | --- | --- |
| **Target emotion** | **Selected emotion** | | | | | | | | | | | |
|  | **Pri** | **Irr** | **Hap** | **Ang** | **Ple** | **Dis** | **Int** | **Anx** | **Rel** | **Fea** | **Sad** | **Des** |
| **PSD+AGG** | | | | | | | | | | | | |
| Pri | **35** | 4 | 37 | 1 | 7 | 1 | 6 | 3 | 4 | 1 | 0 | 1 |
| Irr | 3 | **18** | 1 | 6 | 3 | 3 | 17 | 14 | 20 | 4 | 5 | 6 |
| Hap | 8 | 0 | **53** | 4 | 5 | 2 | 6 | 5 | 6 | 5 | 1 | 5 |
| Ang | 3 | 12 | 1 | **59** | 1 | 6 | 3 | 3 | 1 | 6 | 1 | 5 |
| Ple | 5 | 1 | 36 | 1 | **42** | 0 | 0 | 1 | 12 | 0 | 1 | 1 |
| Dis | 1 | 4 | 3 | 4 | 3 | **42** | 1 | 8 | 4 | 7 | 18 | 5 |
| Int | 10 | 3 | 2 | 1 | 3 | 1 | **44** | 9 | 11 | 1 | 8 | 8 |
| Anx | 1 | 7 | 3 | 3 | 2 | 3 | 16 | **33** | 7 | 11 | 6 | 8 |
| Rel | 18 | 1 | 8 | 0 | 14 | 1 | 6 | 5 | **37** | 1 | 6 | 3 |
| Fea | 2 | 8 | 1 | 15 | 3 | 5 | 3 | 16 | 2 | **21** | 7 | 16 |
| Sad | 11 | 6 | 3 | 3 | 1 | 2 | 7 | 14 | 18 | 3 | **26** | 8 |
| Des | 1 | 3 | 1 | 8 | 1 | 3 | 4 | 15 | 3 | 9 | 24 | **28** |
| **PSD-AGG** | | | | | | | | | | | | |
| Pri | **42** | 2 | 40 | 3 | 3 | 1 | 5 | 1 | 1 | 0 | 0 | 1 |
| Irr | 3 | **24** | 2 | 6 | 1 | 6 | 17 | 17 | 17 | 0 | 5 | 3 |
| Hap | 3 | 3 | **67** | 3 | 1 | 0 | 6 | 2 | 3 | 3 | 3 | 5 |
| Ang | 0 | 18 | 0 | **59** | 0 | 7 | 3 | 4 | 0 | 4 | 1 | 3 |
| Ple | 6 | 0 | 33 | 1 | **49** | 1 | 1 | 1 | 8 | 0 | 0 | 0 |
| Dis | 0 | 3 | 1 | 2 | 0 | **52** | 2 | 6 | 1 | 2 | 19 | 11 |
| Int | 4 | 6 | 1 | 1 | 1 | 1 | **59** | 9 | 10 | 3 | 1 | 4 |
| Anx | 1 | 7 | 1 | 2 | 1 | 4 | 12 | **38** | 5 | 13 | 4 | 13 |
| Rel | 15 | 1 | 9 | 2 | 15 | 1 | 6 | 6 | **38** | 0 | 6 | 1 |
| Fea | 0 | 5 | 0 | 19 | 0 | 3 | 1 | 24 | 0 | **26** | 8 | 15 |
| Sad | 10 | 3 | 1 | 1 | 1 | 2 | 4 | 11 | 13 | 2 | **40** | 13 |
| Des | 1 | 5 | 0 | 5 | 0 | 1 | 1 | 17 | 1 | 10 | 33 | **26** |
| **HC** | | | | | | | | | | | | |
| Pri | **52** | 3 | 35 | 1 | 1 | 2 | 4 | 0 | 1 | 0 | 0 | 0 |
| Irr | 1 | **44** | 1 | 7 | 2 | 4 | 12 | 13 | 11 | 1 | 1 | 3 |
| Hap | 3 | 1 | **68** | 0 | 2 | 0 | 11 | 2 | 2 | 3 | 1 | 6 |
| Ang | 0 | 18 | 0 | **67** | 0 | 2 | 2 | 1 | 0 | 5 | 0 | 5 |
| Ple | 3 | 0 | 32 | 0 | **53** | 0 | 1 | 0 | 11 | 0 | 0 | 0 |
| Dis | 0 | 1 | 0 | 0 | 0 | **62** | 0 | 9 | 0 | 2 | 18 | 8 |
| Int | 2 | 8 | 1 | 1 | 1 | 1 | **63** | 15 | 5 | 1 | 1 | 2 |
| Anx | 0 | 5 | 0 | 1 | 0 | 2 | 17 | **51** | 0 | 12 | 3 | 9 |
| Rel | 13 | 1 | 8 | 1 | 7 | 3 | 6 | 2 | **53** | 0 | 4 | 2 |
| Fea | 1 | 2 | 0 | 9 | 0 | 1 | 0 | 30 | 0 | **30** | 6 | 23 |
| Sad | 6 | 3 | 0 | 1 | 0 | 3 | 5 | 10 | 7 | 3 | **51** | 10 |
| Des | 1 | 3 | 0 | 5 | 0 | 3 | 0 | 17 | 0 | 9 | 23 | **38** |
| *Note.* Diagonals marked in bold represent percentages of correctly identified emotions: Pri (pride), Irr (irritation), Hap (happiness) Ang (anger), Ple (pleasure), Dis (disgust), Int (interest), Anx (anxiety), Rel (relief), Fea (fear), Sad (sadness), and Des (despair). Green cells represent within-valence misclassifications with frequency above 5%. Yellow cells represent cross-valence misclassifications with frequency above 5%. | | | | | | | | | | | | |

| **Table S2.** Confusion matrix illustrating accuracy in auditory modality in the ERAM test for all three groups. | | | | | | | | | | | | |
| --- | --- | --- | --- | --- | --- | --- | --- | --- | --- | --- | --- | --- |
| **Target  emotion** | **Selected emotion** | | | | | | | | | | | |
|  | **Pri** | **Irr** | **Hap** | **Ang** | **Ple** | **Dis** | **Int** | **Anx** | **Rel** | **Fea** | **Sad** | **Des** |
| **PSD+AGG** | | | | | | | | | | | | |
| Pri | **34** | 11 | 20 | 3 | 4 | 4 | 9 | 3 | 6 | 3 | 1 | 1 |
| Irr | 12 | **20** | 1 | 0 | 7 | 3 | 26 | 6 | 13 | 3 | 5 | 4 |
| Hap | 5 | 3 | **28** | 4 | 3 | 4 | 3 | 9 | 2 | 16 | 5 | 17 |
| Ang | 4 | 16 | 1 | **47** | 0 | 7 | 4 | 6 | 3 | 3 | 3 | 5 |
| Ple | 4 | 1 | 3 | 1 | **55** | 1 | 5 | 5 | 15 | 3 | 3 | 3 |
| Dis | 4 | 4 | 4 | 2 | 10 | **10** | 8 | 11 | 18 | 3 | 18 | 7 |
| Int | 14 | 3 | 9 | 3 | 18 | 3 | **25** | 4 | 15 | 1 | 2 | 3 |
| Anx | 8 | 5 | 4 | 3 | 8 | 3 | 13 | **20** | 20 | 9 | 2 | 6 |
| Rel | 2 | 3 | 5 | 1 | 26 | 3 | 2 | 2 | **42** | 6 | 3 | 6 |
| Fea | 0 | 6 | 2 | 10 | 1 | 5 | 1 | 19 | 1 | **32** | 7 | 16 |
| Sad | 5 | 2 | 4 | 2 | 3 | 2 | 10 | 9 | 18 | 3 | **38** | 4 |
| Des | 1 | 3 | 1 | 6 | 0 | 1 | 3 | 15 | 3 | 13 | 35 | **21** |
| **PSD-AGG** | | | | | | | | | | | | |
| Pri | **38** | 15 | 15 | 10 | 1 | 4 | 7 | 1 | 3 | 2 | 1 | 3 |
| Irr | 10 | **32** | 3 | 6 | 4 | 5 | 19 | 3 | 13 | 1 | 3 | 3 |
| Hap | 0 | 5 | **35** | 5 | 1 | 1 | 1 | 5 | 3 | 21 | 8 | 16 |
| Ang | 3 | 15 | 1 | **63** | 0 | 6 | 1 | 3 | 1 | 3 | 2 | 1 |
| Ple | 2 | 1 | 3 | 1 | **60** | 0 | 6 | 5 | 15 | 2 | 3 | 1 |
| Dis | 4 | 4 | 5 | 1 | 6 | **13** | 7 | 12 | 16 | 3 | 24 | 6 |
| Int | 13 | 1 | 7 | 1 | 16 | 1 | **33** | 1 | 19 | 2 | 3 | 2 |
| Anx | 0 | 4 | 1 | 1 | 16 | 4 | 11 | **25** | 12 | 12 | 8 | 6 |
| Rel | 0 | 2 | 1 | 3 | 32 | 1 | 1 | 3 | **46** | 2 | 1 | 7 |
| Fea | 0 | 3 | 2 | 8 | 0 | 3 | 0 | 17 | 1 | **44** | 3 | 18 |
| Sad | 2 | 1 | 3 | 0 | 3 | 1 | 7 | 17 | 18 | 7 | **35** | 6 |
| Des | 1 | 3 | 1 | 3 | 0 | 3 | 1 | 10 | 2 | 16 | 28 | **30** |
| **HC** | | | | | | | | | | | | |
| Pri | **50** | 10 | 23 | 4 | 3 | 1 | 7 | 0 | 1 | 0 | 0 | 1 |
| Irr | 15 | **41** | 2 | 3 | 1 | 3 | 23 | 3 | 6 | 0 | 1 | 2 |
| Hap | 3 | 1 | **35** | 3 | 1 | 1 | 1 | 5 | 1 | 22 | 3 | 24 |
| Ang | 1 | 29 | 1 | **59** | 1 | 3 | 2 | 2 | 0 | 1 | 0 | 1 |
| Ple | 2 | 0 | 1 | 0 | **70** | 0 | 5 | 5 | 10 | 0 | 5 | 2 |
| Dis | 3 | 8 | 1 | 1 | 9 | **18** | 6 | 12 | 10 | 1 | 19 | 14 |
| Int | 10 | 2 | 6 | 0 | 20 | 1 | **44** | 2 | 13 | 0 | 1 | 0 |
| Anx | 2 | 6 | 1 | 1 | 10 | 2 | 9 | **34** | 12 | 13 | 5 | 6 |
| Rel | 0 | 2 | 1 | 0 | 37 | 1 | 0 | 3 | **54** | 1 | 0 | 2 |
| Fea | 0 | 4 | 1 | 4 | 0 | 1 | 0 | 17 | 0 | **56** | 1 | 16 |
| Sad | 1 | 1 | 2 | 0 | 1 | 1 | 7 | 11 | 12 | 3 | **53** | 9 |
| Des | 2 | 1 | 1 | 1 | 1 | 1 | 0 | 15 | 0 | 8 | 38 | **33** |
| *Note.* Diagonals marked in bold represent percentages of correctly identified emotions: Pri (pride), Irr (irritation), Hap (happiness) Ang (anger), Ple (pleasure), Dis (disgust), Int (interest), Anx (anxiety), Rel (relief), Fea (fear), Sad (sadness), and Des (despair). Green cells represent within-valence misclassifications with frequency above 5%. Yellow cells represent cross-valence misclassifications with frequency above 5%. | | | | | | | | | | | | |

| **Table S3.** Confusion matrix illustrating accuracy in multimodal presentation in the ERAM test for all three groups. | | | | | | | | | | | | |
| --- | --- | --- | --- | --- | --- | --- | --- | --- | --- | --- | --- | --- |
| **Target  emotion** | **Selected emotion** | | | | | | | | | | | |
|  | **Pri** | **Irr** | **Hap** | **Ang** | **Ple** | **Dis** | **Int** | **Anx** | **Rel** | **Fea** | **Sad** | **Des** |
| **PSD+AGG** | | | | | | | | | | | | |
| Pri | **46** | 1 | 14 | 1 | 4 | 0 | 13 | 2 | 18 | 0 | 1 | 2 |
| Irr | 8 | **26** | 3 | 2 | 3 | 6 | 22 | 11 | 9 | 4 | 3 | 4 |
| Hap | 8 | 1 | **59** | 1 | 7 | 1 | 1 | 3 | 12 | 3 | 1 | 4 |
| Ang | 1 | 16 | 1 | **58** | 0 | 6 | 3 | 4 | 0 | 6 | 2 | 4 |
| Ple | 3 | 1 | 25 | 2 | **51** | 0 | 2 | 1 | 13 | 0 | 1 | 1 |
| Dis | 1 | 5 | 2 | 4 | 1 | **49** | 1 | 9 | 3 | 4 | 15 | 8 |
| Int | 11 | 3 | 15 | 0 | 8 | 0 | **46** | 3 | 8 | 1 | 4 | 0 |
| Anx | 3 | 4 | 3 | 2 | 1 | 2 | 13 | **37** | 10 | 11 | 8 | 6 |
| Rel | 14 | 1 | 12 | 0 | 13 | 1 | 3 | 2 | **49** | 2 | 1 | 3 |
| Fea | 1 | 7 | 3 | 3 | 2 | 1 | 6 | 28 | 3 | **37** | 2 | 8 |
| Sad | 2 | 8 | 4 | 3 | 4 | 4 | 10 | 15 | 13 | 5 | **24** | 8 |
| Des | 0 | 3 | 5 | 1 | 2 | 1 | 4 | 20 | 4 | 8 | 25 | **27** |
| **PSD-AGG** | | | | | | | | | | | | |
| Pri | **42** | 2 | 13 | 0 | 6 | 0 | 17 | 0 | 16 | 1 | 1 | 1 |
| Irr | 4 | **27** | 4 | 5 | 2 | 6 | 25 | 12 | 5 | 1 | 1 | 8 |
| Hap | 3 | 1 | **68** | 0 | 3 | 0 | 0 | 1 | 17 | 0 | 3 | 3 |
| Ang | 1 | 13 | 0 | **76** | 0 | 3 | 0 | 3 | 0 | 1 | 2 | 2 |
| Ple | 6 | 0 | 24 | 0 | **53** | 0 | 1 | 0 | 15 | 0 | 1 | 0 |
| Dis | 0 | 6 | 1 | 3 | 0 | **56** | 2 | 4 | 0 | 6 | 13 | 10 |
| Int | 14 | 1 | 13 | 0 | 8 | 1 | **48** | 0 | 10 | 2 | 1 | 3 |
| Anx | 0 | 1 | 1 | 3 | 2 | 1 | 4 | **58** | 8 | 13 | 3 | 6 |
| Rel | 11 | 0 | 8 | 1 | 16 | 0 | 3 | 1 | **59** | 0 | 2 | 0 |
| Fea | 0 | 4 | 0 | 2 | 1 | 1 | 4 | 31 | 1 | **46** | 3 | 7 |
| Sad | 1 | 5 | 1 | 0 | 2 | 4 | 2 | 17 | 8 | 4 | **40** | 16 |
| Des | 0 | 2 | 1 | 2 | 1 | 1 | 1 | 17 | 2 | 6 | 37 | **31** |
| **HC** | | | | | | | | | | | | |
| Pri | **51** | 1 | 14 | 1 | 2 | 0 | 16 | 1 | 14 | 0 | 0 | 0 |
| Irr | 3 | **50** | 1 | 2 | 0 | 6 | 20 | 9 | 3 | 1 | 1 | 4 |
| Hap | 4 | 0 | **73** | 0 | 2 | 0 | 0 | 1 | 21 | 0 | 0 | 0 |
| Ang | 0 | 17 | 0 | **79** | 0 | 0 | 0 | 2 | 0 | 2 | 0 | 1 |
| Ple | 2 | 0 | 22 | 0 | **61** | 1 | 1 | 0 | 13 | 0 | 1 | 0 |
| Dis | 0 | 3 | 1 | 2 | 0 | **70** | 0 | 2 | 0 | 1 | 15 | 6 |
| Int | 14 | 0 | 11 | 0 | 5 | 0 | **61** | 1 | 6 | 0 | 0 | 2 |
| Anx | 0 | 1 | 0 | 0 | 1 | 2 | 3 | **66** | 3 | 15 | 6 | 3 |
| Rel | 14 | 0 | 17 | 0 | 10 | 0 | 2 | 0 | **57** | 0 | 1 | 0 |
| Fea | 0 | 2 | 0 | 1 | 0 | 0 | 1 | 29 | 1 | **61** | 0 | 5 |
| Sad | 0 | 4 | 0 | 1 | 0 | 10 | 1 | 17 | 5 | 2 | **46** | 14 |
| Des | 1 | 1 | 0 | 2 | 0 | 2 | 1 | 15 | 1 | 5 | 27 | **47** |
| *Note.* Diagonals marked in bold represent percentages of correctly identified emotions: Pri (pride), Irr (irritation), Hap (happiness) Ang (anger), Ple (pleasure), Dis (disgust), Int (interest), Anx (anxiety), Rel (relief), Fea (fear), Sad (sadness), and Des (despair). Green cells represent within-valence misclassifications with frequency above 5%. Yellow cells represent cross-valence misclassifications with frequency above 5%. | | | | | | | | | | | | |

| **Table S4.** Means (Hu), standard deviations and pairwise *t*-tests (*p*-values) of individual emotions across all three participant groups | | | | | | | | | | | | | |
| --- | --- | --- | --- | --- | --- | --- | --- | --- | --- | --- | --- | --- | --- |
|  |  | Pairwise *t*-tests (*df* = 236) | | | | | | | | | | | |
| Emotion | *M (SD)* | Pri | Irr | Hap | Ang | Ple | Dis | Int | Anx | Rel | Fea | Sad | Des |
| Pri | .26 (.18) |  | **6.45** | 1.73 (.09) | **13.34** | **5.98** | **3.68** | .25 (.81) | **7.53** | 1.70 (.09) | 1.85 (.07) | **5.59** | **7.46** |
| Irr | .17 (.18) |  |  | **7.52** | **20.91** | **12.49** | **9.25** | **6.57** | .90 (.33) | **5.08** | **4.54** | 1.04 (.30) | 1.12 (.27) |
| Hap | .28 (.17) |  |  |  | **12.23** | **4.43** | 2.39 (.02) | 1.67 (.10) | **8.91** | **3.37** | **3.55** | **6.97** | **8.83** |
| Ang | .50 (.26) |  |  |  |  | **9.23** | **9.59** | **14.46** | **21.16** | **14.84** | **15.33** | **17.93** | **20.05** |
| Ple | .35 (.21) |  |  |  |  |  | 1.62 (.11) | **6.68** | **14.24** | **8.56** | **7.44** | **11.32** | **12.90** |
| Dis | .32 (.23) |  |  |  |  |  |  | **3.88** | **10.08** | **5.42** | **5.22** | **9.06** | **10.20** |
| Int | .16 (.21) |  |  |  |  |  |  |  | **7.68** | 1.42 (.16) | 1.53 (.13) | **5.06** | **6.72** |
| Anx | .16 (.15) |  |  |  |  |  |  |  |  | **6.06** | **5.94** | 1.86 (.07) | .34 (.74) |
| Rel | .24 (.18) |  |  |  |  |  |  |  |  |  | 0.20 (.84) | **3.92** | **5.88** |
| Fea | .23 (.20) |  |  |  |  |  |  |  |  |  |  | **3.59** | **5.83** |
| Sad | .19 (.17) |  |  |  |  |  |  |  |  |  |  |  | 2.40 (.02) |
| Des | .16 (.17) |  |  |  |  |  |  |  |  |  |  |  |  |
| *Note.* Exact *p*-values only provided when different from < .001. Values marked in bold indicate differences that remained significant after Holm-Bonferroni correction (*p* < .05). Pri (pride), Irr (irritation), Hap (happiness) Ang (anger), Ple (pleasure), Dis (disgust), Int (interest), Anx (anxiety), Rel (relief), Fea (fear), Sad (sadness), and Des (despair). | | | | | | | | | | | | | |

| **Table S5.** Mixed analyses of variance (ANOVA) with gender as an additional between-subjects variable. | | | | | | |
| --- | --- | --- | --- | --- | --- | --- |
|  | ***SS*** | ***df*** | ***MS*** | ***F*** | ***p*** | ***η*^2^*_p_*** |
| **ANOVA 1** | | | | | | |
| Group | 1.90 | 2 | 0.95 | 27.81 | < .001 | .19 |
| Gender | 0.14 | 1 | 0.14 | 3.94 | .05 | .02 |
| Group × Gender | 0.06 | 2 | 0.03 | 0.80 | .45 | .01 |
| Error | 7.90 | 231 | 0.03 |  |  |  |
| Modality^a^ | 1.88 | 1.98 | 0.95 | 107.57 | < .001 | .32 |
| Modality × Group^a^ | 0.06 | 3.95 | 0.02 | 1.70 | .15 | .01 |
| Modality × Gender^a^ | 0.09 | 1.98 | 0.05 | 5.14 | .01 | .02 |
| Modality × Group × Gender^a^ | 0.04 | 3.95 | 0.01 | 1.05 | .38 | .01 |
| Error (modality)^a^ | 4.03 | 456.07 | 0.01 |  |  |  |
| **ANOVA 2** | | | | | | |
| Group | 7.15 | 2 | 3.58 | 26.65 | < .001 | .19 |
| Gender | 0.46 | 1 | 0.46 | 3.42 | .07 | .02 |
| Group × Gender | 0.35 | 2 | 0.17 | 1.30 | .28 | .01 |
| Error | 30.98 | 231 | 0.13 |  |  |  |
| Emotion^a^ | 17.93 | 9.92 | 1.81 | 65.08 | < .001 | .22 |
| Emotion × Group^a^ | 0.52 | 19.84 | 0.03 | 0.94 | .54 | .01 |
| Emotion × Gender^a^ | 0.61 | 9.92 | 0.06 | 2.22 | .02 | .01 |
| Emotion × Group × Gender^a^ | 0.59 | 19.84 | 0.03 | 1.07 | .38 | .01 |
| Error (emotion)^a^ | 63.64 | 2291.68 | 0.03 |  |  |  |
| **ANOVA 3** | | | | | | |
| Group | 2.38 | 2 | 1.19 | 26.52 | < .001 | .19 |
| Gender | 0.15 | 1 | 0.15 | 2.25 | .06 | .01 |
| Group × Gender | 0.12 | 2 | 0.06 | 1.33 | .27 | .01 |
| Error | 10.36 | 231 | 0.05 |  |  |  |
| Valence | 0.00 | 1 | 0.00 | 15.38 | < .001 | .06 |
| Valence × Group | 0.00 | 2 | 0.00 | 0.54 | .58 | .01 |
| Valence × Gender | 0.00 | 1 | 0.00 | 0.13 | .71 | .00 |
| Valence × Group × Gender | 0.00 | 2 | 0.00 | 0.62 | .54 | .01 |
| Error (valence) | 0.01 | 231 | 0.00 |  |  |  |
| Arousal | 0.44 | 1 | 0.44 | 120.93 | < .001 | .34 |
| Arousal × Group | 0.01 | 2 | 0.00 | 0.84 | .43 | .01 |
| Arousal × Gender | 0.00 | 1 | 0.00 | 0.06 | .81 | .00 |
| Arousal × Group × Gender | 0.00 | 2 | 0.00 | 0.58 | .56 | .01 |
| Error (arousal) | 0.84 | 231 | 0.00 |  |  |  |
| Valence × Arousal | 0.07 | 1 | 0.07 | 10.85 | .001 | .05 |
| Valence × Arousal × Group | 0.00 | 2 | 0.00 | 0.26 | .77 | .00 |
| Valence × Arousal × Gender | 0.00 | 1 | 0.00 | 0.40 | .53 | .00 |
| Valence × Arousal × Group × Gender | 0.01 | 2 | 0.00 | 0.50 | .61 | .00 |
| Error (valence × arousal) | 1.51 | 231 | 0.01 |  |  |  |
| *Note*. ANOVA 1: 3 (group) × 2 (gender) × 3 (modality). ANOVA 2: 3 (group) × 2 (gender) × 12 (emotion). ANOVA 3: 3 (group) × 2 (gender) × 2 (valence) × 2 (arousal).  ^a^ = Huynh-Feldt correction, *SS* = Type III Sum of Squares, *MS* = Mean Square | | | | | | |

| **Table S6.** Bivariate correlations between overall emotion recognition (Hu), clinical, cognitive and sociodemographic characteristics for the PSD groups. Pearson correlations (*r*) are shown in the upper triangle and Spearman correlations (*ρ*) are shown in the lower triangle. | | | | | | | | | | | | | |
| --- | --- | --- | --- | --- | --- | --- | --- | --- | --- | --- | --- | --- | --- |
|  | ***N*** | **1.** | **2.** | **3.** | **4.** | **5.** | **6.** | **7.** | **8.** | **9.** | **10.** | **11.** | **12.** |
| 1. Emotion recognition | 151 |  | -.09 | -.21** | .30*** | .55*** | -.05 | -.25** | -.08 | .28*** | .47*** | .26*** | .10 |
| 2. Illness duration | 151 | -.12 |  | .03 | .14 | -.10 | -.20* | .08 | .15 | -.16* | -.02 | .21** | .20* |
| 3. Antipsychotic dose/day | 151 | -.31*** | .09 |  | -.24* | -.17* | .28*** | .20* | .21** | -.14 | -.10 | -.27*** | -.01 |
| 4. Education | 151 | .30*** | .11 | -.28*** |  | .25** | -.23** | -.18* | -.38 | .16* | .06 | .39*** | -.06 |
| 5. Fluid intelligence | 143 | .55*** | -.10 | -.29*** | .24** |  | -.16 | -.19* | -.12 | .36*** | .41*** | .21* | -.16 |
| 6. Prior AUD/SUD | 151 | -.07 | -.20* | .27*** | -.23** | -.15 |  | -.04 | -.01 | .11 | -.10 | -.43*** | -.15 |
| 7. SANS | 151 | -.20* | .10 | .18* | -.18* | -.18* | -.01 |  | .10 | -.20* | -.16 | -.13 | -.05 |
| 8. SAPS | 151 | -.10 | .26** | .23** | -.08 | -.16 | -.01 | .25** |  | .02 | -.08 | .08 | .20* |
| 9. Psychomotor speed | 150 | .28*** | -.20* | -.19* | .15 | .37*** | .10 | -.23** | -.01 |  | .12 | .10 | -.13 |
| 10. Synonym test | 145 | .46*** | -.02 | -.16 | .08 | .40*** | -.05 | -.07 | -.13 | .04 |  | -.01 | .10 |
| 11. Group | 151 | .27*** | .20* | -.32*** | .39*** | .20* | -.43*** | -.15 | .06 | .13 | -.01 |  | .07 |
| 12. Gender | 151 | .08 | .21* | -.02 | -.06 | -.14 | -.14 | -.01 | .17* | -.15 | .10 | .07 |  |
| *Note.* Education (compulsory/primary schooling only = 0, upper secondary education or higher = 1); Prior AUD (alcohol use disorder)/SUD (substance use disorder) (no = 0, yes = 1); SANS (Scale for the Assessment of Negative Symptoms); SAPS (Scale for the Assessment of Positive Symptoms); Group (PSD+AGG = 0, PSD-AGG = 1); Gender (male = 0, female = 1). *N* = 137–151.  Non-normally distributed data: Illness duration, Antipsychotic dose/day, SAPS, Synonym test.  * *p* < .05, ** *p* < .01, *** *p* < .001, Sig. (2–tailed) | | | | | | | | | | | | | |
